# External validation of anti-Müllerian hormone based prediction of live birth in assisted conception

**DOI:** 10.1186/1757-2215-6-3

**Published:** 2013-01-07

**Authors:** Amani Khader, Suzanne M Lloyd, Alex McConnachie, Richard Fleming, Valentina Grisendi, Antonio La Marca, Scott M Nelson

**Affiliations:** 1 School of Medicine, University of Glasgow, Glasgow, G12 8QQ, UK; 2 Robertson Centre for Biostatistics, University of Glasgow, Glasgow, G12 8QQ, UK; 3 Glasgow Centre for Reproductive Medicine, Glasgow, G51 4FD, UK; 4 Mother-Infant Department, Institute of Obstetrics and Gynecology, University of Modena and Reggio Emilia, Modena, 41100, Italy

**Keywords:** AMH, Live birth prediction, IVF

## Abstract

**Background:**

Chronological age and oocyte yield are independent determinants of live birth in assisted conception. Anti-Müllerian hormone (AMH) is strongly associated with oocyte yield after controlled ovarian stimulation. We have previously assessed the ability of AMH and age to independently predict live birth in an Italian assisted conception cohort. Herein we report the external validation of the nomogram in 822 UK first in vitro fertilization (IVF) cycles.

**Methods:**

Retrospective cohort consisting of 822 patients undergoing their first IVF treatment cycle at Glasgow Centre for Reproductive Medicine. Analyses were restricted to women aged between 25 and 42 years of age. All women had an AMH measured prior to commencing their first IVF cycle. The performance of the model was assessed; discrimination by the area under the receiver operator curve (ROC_AUC_) and model calibration by the predicted probability versus observed probability.

**Results:**

Live births occurred in 29.4% of the cohort. The observed and predicted outcomes showed no evidence of miscalibration (p = 0.188). The ROC_AUC_ was 0.64 (95% CI: 0.60, 0.68), suggesting moderate and similar discrimination to the original model. The ROC_AUC_ for a continuous model of age and AMH was 0.65 (95% CI 0.61, 0.69), suggesting that the original categories of AMH were appropriate.

**Conclusions:**

We confirm by external validation that AMH and age are independent predictors of live birth. Although the confidence intervals for each category are wide, our results support the assessment of AMH in larger cohorts with detailed baseline phenotyping for live birth prediction.

## Background

Chronological age and oocyte yield are independent determinants of live birth in assisted conception [1]. Circulating anti-Müllerian hormone (AMH) levels are strongly associated with oocyte yield after controlled ovarian stimulation [2]. This strong correlation with oocyte yield underlies the independent associations of AMH and age with live birth, which has now been confirmed in several studies [3-7]. We previously exploited this relationship to construct a nomogram for the prediction of live birth using a combination of age and AMH in an Italian cohort of 381 IVF cycles [8].

In the original study (8), 101 of 381 women (26.5%) achieved a live birth. Univariate and multivariate logistic regression revealed significant decreasing odds of live birth for increasing age and decreasing AMH irrespective of whether they were treated as continuous or our predestined categorical variables. In order to facilitate the practical use of this model, a single 3×3 table was developed (Table 1). By cross tabulating any given patients age with their AMH level, the probability of the live birth following IVF may be easily calculated. For example according to the nomogram, a woman aged 31–37 years and with a serum AMH levels of 0.4-2.8 ng/ml has a 27% probability of achieving a live birth with a confidence interval varying from 21% to 35%. Evidence of the independent effects of age and AMH can be seen examining the relative chance of success by comparing categories vertically and horizontally respectively. For example for the same AMH category of 0.4-2.8 ng/ml women in the age category >37 years had a chance of live birth that was 52% lower than women who were <31 years old. Similarly women who were <31 years old but with very low AMH levels (<0.4 ng/ml) had a chance of live birth decreased by 75% when compared with women of the same age but with a high AMH level (> 2.8 ng/ml).

**Table 1:** The prediction model for a live birth based on age and AMH (with permission from ref.8)

**Age (years)**		**AMH (ng/ml)**	
	<0.4	0.4- < 2.8	≥2.8
<31	0.13 (0.04–0.36)	0.38 (0.26–0.51)	0.52 (0.38–0.67)
31–37	0.09 (0.02–0.24)	0.27 (0.21–0.35)	0.40 (0.28–0.54)
>37	0.05 (0.01–0.16)	0.18 (0.12–0.26)	0.29 (0.17–0.44)

For each AMH and age category the probability (95% CI) of live birth after IVF is indicated.

At present although a variety of prediction models have been reported, we are not aware of any other models that incorporate AMH for the prediction of live birth in IVF cycles (9). Our AMH-age model therefore has the potential to become a clinically useful addition to the fertility workup of infertile couples. However before clinicians can adopt any prediction models into routine clinical practice, the accuracy of the model should be independently evaluated in a population different from the one on which the model was elaborated [9,10]. External validation (EV) of the discriminative power and calibration of the model is therefore crucial to assess the generalizability of our model to other populations. The objective of the present study was to validate our previously developed prediction model for the live birth in IVF cycles in an independent large cohort of infertile women.

## Methods

### Validation cohort profile

This study analysed the database containing the clinical and laboratory information on IVF treatment cycles carried out at Glasgow Centre for Reproductive Medicine, Glasgow 2006 – 2010. These data were collected prospectively and recorded in the registered database in the fertility centre in Glasgow, UK. Patients were stimulated in accordance with our previously published policies using a combination of agonist and antagonist strategies based on ovarian reserve assessment. For this analysis cycles were selected for analysis if they were the first IVF/ICSI cycle. We had previously limited our analyses to women aged between 25 and 42 years of age (8) and we censored the EV dataset in keeping with our previous age restriction. Embryo transfer policy was in line with UK regulations with predominantly two embryos transferred in women <40 and three in woman ≥40 years. Live birth was defined as at least one infant born alive after 24 weeks gestation, consistent with previous prediction models and publications.

### AMH analysis

AMH was measured prior to commencement of all IVF cycles and was measured on any day of the cycle. The AMH assay used was the commercial ELISA kit provided by DSL(Webster, TX, USA), with values initially presented in concentrations of picomoles per litre (conversion factor to pmol/l = ng/ml × 7.143). Inter and intra-assay coefficients of variation were 5.3 and 5.4%, respectively. The development cohort had utilised the Immunotech assay and therefore the EV cohort values were transformed using our previously reported equation AMH Immunotech = 1.40 DSL–0.62 pmol/L, with subsequent conversion to ng/ml (12). As AMH was not normally distributed it was log transformed in accordance with previous analyses.

### Statistical analysis

Validation of a prediction model comprises two characteristics of diagnostic performance: calibration which is the agreement between predictions and observations in the validation cohort and discrimination which is the ability of the model to distinguish between women with or without live birth.

The subjects were split into the same nine groups based on age and logged-AMH values as previously reported for the development cohort. The groups were defined as follows:

1. Age > 37 & AMH < 0.4

2. Age > 37 & 0.4 ≤ AMH < 2.8

3. Age > 37 & AMH ≥ 2.8

4. 31 ≤ Age ≤ 37 & AMH < 0.4

5. 31 ≤ Age ≤ 37 & 0.4 ≤ AMH < 2.8

6. 31 ≤ Age ≤ 37 & AMH ≥ 2.8

7. Age < 31 & AMH < 0.4

8. Age < 31 & 0.4 ≤ AMH < 2.8

9. Age < 31 & AMH ≥ 2.8

The predicted and observed probability of live birth for each group were derived using the respective formulae (8):

$$P_{\text{pred}}\left(\text{live}\;\text{birth}\right)=\frac{\text{exp}(-2.88+1.38*\text{lnAM}H_{0.4-2.8}+1.96*\text{lnAM}{H_{>}}_{2.8}+1.01*\text{Ag}e_{<31}+0.52*\text{Ag}e_{31-37}}{1+\text{exp}(-2.88+1.38*\mathrm{lnAM}H_{0.4-2.8}+1.96*\text{lnAM}{H_{>}}_{2.8}+1.01*\text{Ag}e_{<31}+0.52*\text{Ag}e_{31-37}}$$

$$P_{\text{obs}}\left(\text{live}\;\text{birth}\right)=\frac{\text{Number}\;\text{of}\;\text{live}\;\text{births}\;\text{per}\;\text{group}}{\text{Total}\;\text{number}\;\text{of}\;\text{subjects}\;\text{per}\;\text{group}}$$

The predicted number of live births was calculated for each group by multiplying the predicted probability by the total number of subjects in the group. The predicted number of women without a live birth was calculated as the total number of subjects per group minus the predicted number of live births. Groups were pooled to allow expected counts to have >5 subjects, with comparison of observed and predicted assessed by chi-squared test.

A. Age > 37 & AMH < 2.8

B. Age > 37 & AMH ≥ 2.8

C. 31 ≤ Age ≤ 37 & AMH < 2.8

D. 31 ≤ Age ≤ 37 & AMH ≥ 2.8

E. Age < 31 & AMH < 2.8

F. Age < 31 & AMH ≥ 2.8

The discrimination of the model was assessed by the area under the receiver operator curve (ROC_AUC_), and the calibration by the predicted probability versus observed probability. A logistic regression model was fitted with age and AMH as continuous variables; the discrimination of this model was compared to that from the model with age and AMH as categories to investigate the relative accuracy of the age and AMH cut-offs used in the nomogram. Analyses were performed using SASv9.2.

## Results

A total of 822 patients were selected on the basis of inclusion criteria and baseline clinical characteristics and treatment outcomes are provided in Table 2. For convenience the original development cohort details are also provided. Age was similar to the development cohort, with AMH being significantly higher and duration of infertility shorter in the EV cohort. There were differences also in the causes of infertility as more patients with endometriosis or ovulatory disturbances were included in the development cohort. For the reported outcomes, the EV cohort had significantly shorter duration of stimulation but the number of oocytes per patient was the same in the two groups. The number of patients with an embryo transfer and the number of embryos transferred were both higher in the development cohort, however, clinical pregnancy and live birth rates were similar (Table 2).

**Table 2:** Summary of baseline characteristics and treatment outcomes for development and validation cohort

**Characteristic**	**Development cohort**	**Validation cohort**	***P***
	**N = 381**	**N = 822**	
Age (years)	34.8 +/− 4.48	35.3 +/− 4.28	Ns
BMI (kg/m^2^)	24 +/− 5.8	24.6 +/− 4.30	<.05
AMH (ng/ml)	1.3 (0.03, 13.8)	2.16 (0.02, 38.6)	<.05
Duration of infertility (years)	2.8 +/− 1.7	2.49 +/− 2.36	<.05
Cause of infertility			
Combination of cause	67 (17.5%)	71 (8.6%)	<.05
Endometriosis	45 (11.8%)	39 (4.7%)	<.05
Idiopathic	140 (36.8%)	270 (32.8%)	Ns
Male factor	123 (32.4%)	285 (34.7%)	Ns
Ovulatory	82 (21.5%)	35 (4.3%)	<.05
Tubal disease	57 (15.0%)	122 (14.8%)	Ns
Treatment outcomes:			
Duration of stimulation (days)	12.8 +/− 2.8	10.4 +/− 2.11	<.05
Dose (IU)	205 +/− 58.6	216 +/− 59.0	<.05
Oocytes per patient	8.5 +/− 5.1	7.99 +/− 4.71	Ns
Embryo transfers performed	347 (91.1%)	713 (86.7%)	<.05
Number of embryos transferred:			
0	34 (9.8%)	109 (13.3%)	Ns
1	8 (2.3%)	127 (15.5%)	<.05
2	83 (23.9%)	534 (65.0%)	<.05
3	256 (73.8%)	52 (6.3%)	<.05
Clinical pregnancy	127 (33.3%)	264 (32.1%)	Ns
Live birth	101 (26.5%)	242 (29.4%)	Ns

Values are presented as mean +/− SD, median (range) or n (%).

Ns: non-significant.

**Figure 1:**
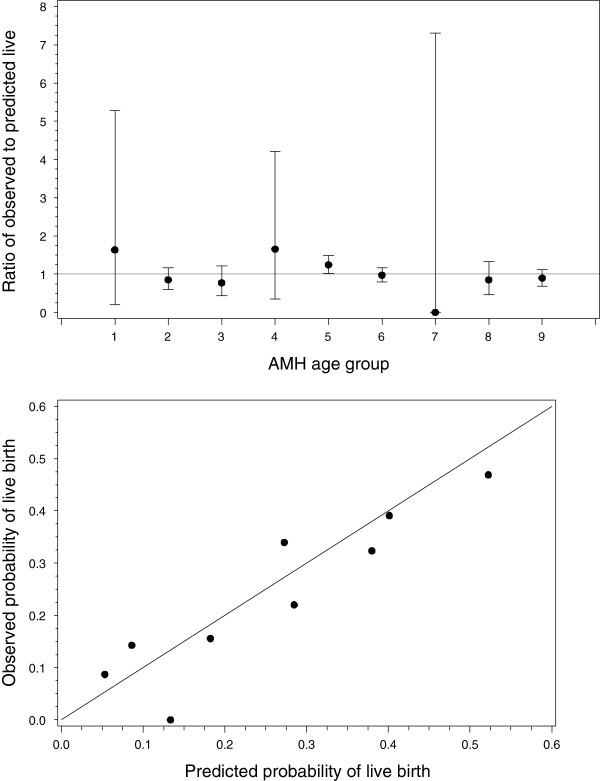
**Top panel: Plot of observed/predicted probabilities of a live birth with 95% confidence intervals.** No data met the criteria for group seven. Lower panel: Calibration plot of the predicted probability versus observed probability of live birth for the nine groups, with a line of equality. In the figure the point corresponding to zero refers to the category for which no patients met the appropriate age and AMH criteria.

Predicted and observed outcomes are shown in Table 3. There was no evidence of a difference in the observed and predicted probabilities of live birth (p =0.188, χ^2^ with 6 degrees of freedom). A plot of the ratio of observed to predicted probabilities demonstrated that all confidence intervals included the null, confirming that there were no significant differences (Figure 1); the calibration plot similarly suggests good calibration of the model (Figure 1). The ROC_AUC_ was 0.64 (95% CI: 0.60, 0.68), suggesting only moderate discriminative ability, similar to the original value of 0.66 (95%CI: 0.61, 0.72)(8).

**Table 3:** Expected probability of a live birth based on La Marca model versus observed live birth

		***Probability of live birth***		***Live birth***		***No live birth***		
***Age-AMH Group***	***Covariate pattern***	***Observed***	***Predicted***	***Observed***	***Expected***	***Observed***	***Expected***	***Total***
1	Age > 37; AMH < 0.4	0.0870	0.0532	2	1.22	21	21.78	23
2	Age > 37; AMH 0.4 - 2.8	0.1557	0.1824	33	38.67	179	173.3	212
3	Age > 37; AMH > = 2.8	0.2203	0.2850	13	16.81	46	42.19	59
4	Age 31–37; AMH < 0.4	0.1429	0.0863	3	1.81	18	19.19	21
5	Age 31–37; AMH 0.4 - 2.8	0.3396	0.2729	72	57.85	140	154.1	212
6	Age 31–37; AMH > = 2.8	0.3911	0.4013	70	71.83	109	107.2	179
7	Age < 31; AMH < 0.4	0.0000	0.1335	0	0.13	1	0.87	1
8	Age < 31; AMH 0.4 - 2.8	0.3235	0.3799	11	12.92	23	21.08	34
9	Age < 31; AMH > = 2.8	0.4691	0.5225	38	42.32	43	38.68	81

Logistic regression for live birth with respect to age and AMH, both fitted as continuous variables, demonstrated decreased odds with increasing age (odds ratio (OR) 0.91, 95% CI 0.87,0.94; p ≤ 0.0001), and a trend for increased odds with higher AMH levels (OR 1.04, 95% CI 0.99,1.09; p = 0.1297). The ROC_AUC_ for a continuous model of age and AMH was similar at 0.65 (95% CI 0.61, 0.69), suggesting no disadvantage to the cut-offs employed in the nomogram.

## Discussion

This study externally validates our AMH-age based prediction of live birth for IVF [8]. Furthermore equivalent model performance was demonstrated in the EV cohort, with confirmation of the independent associations of AMH and age with live birth [3-7].

Recent literature has identified an array of factors which can influence the success of ART, with various prediction models utilizing these factors to aid the determination of a couple’s likelihood of success [11-13]. However, the use of such prediction models clinically has remained limited, largely due to lack of external validation. Of the 29 pregnancy prediction models identified in a recent systematic review, only 8 were externally validated, with only 3 of these applicable to IVF [11]. Our model adds to this literature, allowing stratification of the probability of live birth prior to the commencement of treatment. A relevant difference with previous published model of live birth in IVF is that while the majority of prediction models are based on variables measured during the IVF cycle (e.g. number and quality of embryos), the AMH-age model is based on only baseline characteristics, hence permitting it to be used by clinicians and patients prior to commencing stimulation.

A criticism of the original study was the cut off points given to age and AMH levels in the nomogram and the potential for predictive power of the model to be attenuated by these designated cut-offs. In order to overcome this potential weakness, we additionally investigated the use of age and AMH as continuous variables. The ROC_AUC_ achieved through this mechanism was 0.65, which was identical to that achieved originally, suggesting that the use of the proposed cut-offs does not compromise the predicted probabilities generated and that alternative values would not improve predictions. This is reassuring and allows AMH and age to be displayed as categories, rather continuous variables, in tables. This has clear benefit for applying the model in a clinical environment, with simple cross tabulation of the patient’s age with their AMH concentration rather than having to apply a complex logistic regression formula.

In the EV cohort the discriminative ability of the model was only moderate (ROC_AUC_: 0.66), meaning that the model has limited capacity to be able to correctly distinguish between women who will or will not have a baby following IVF. However, ROC curves are primarily designed for diagnostic models (15), rather for prognostic models accuracy is better assessed by examining calibration (16). Calibration is evaluated by determining the level of correspondence between the calculated pregnancy probabilities and the observed proportion of pregnancies. A well-calibrated model for IVF would be able to classify individuals into whether they have a low, medium or high probability of achieving a live birth. In contrast to the relatively modest discrimination, the calibration of the model was found to be good (Figure 1).

The strength of this study is that the sample size was more than twice that used for model derivation. However, the EV cohort differed from the original cohort for several characteristics such as BMI and duration of infertility and also the intermediate outcome of IVF were different between the two cohorts. This largely reflects the difference existing between the demographic characteristics of Italian and Scottish infertility populations and also the different IVF clinical practices between the two countries. Particularly as the initial study was undertaken when the Italian law regulating assisted reproduction limited the number of inseminated oocytes to three, thereby reducing the number of embryos that may be generated for each patient, was still operative. This resulted in a discordance in the number of embryos transferred, with the all available embryos being transferred in Modena – mainly three; while single or double embryo transfer dominated in Glasgow. In the EV cohort, women were included irrespective of the cause of infertility, past medical history or type of stimulation. Despite these relatively important differences in patient characteristics, legislation and clinical practice the proposed model still fitted very well, further highlighting the potential generalizability of the prognostic model.

The original study limited its analysis to age and AMH, as only these two factors were identified as predictive in the original multivariate analysis for model development (8). We are aware that additional characteristics including BMI, cause and duration of infertility may influence results and the lack of association of these baseline factors with live birth, may have reflected the size of the original cohort (14).

Finally it should be acknowledged that the probabilities generated have relatively wide confidence intervals for all groups; therefore a couple’s predicted likelihood can range significantly. For example, women aged below 31, with AMH levels less than 0.4 ng/mL, are predicted a 13% chance of live birth, however, the confidence interval ranges from 4 to 36% which does not infer much reassurance in their chances of successful outcome. It would however be inappropriate to withhold treatment purely based on the probability estimates derived from our nomogram [14]. Even in women with an AMH below or close to the functional sensitivity of the assay, natural and assisted conception pregnancies have been reported [15-18]. Therefore clinical consultations would require interplay of both the interpretation of the nomogram results by the clinician and individual patient opinion as to whether the probabilities produced could be of benefit. The greatest utility of this external validation may therefore be to confirm that AMH is an independent predictor of live birth and is worthy of evaluation in larger cohorts with detailed baseline phenotyping, with a view to assessing its utility in improving model performance [13,19].

## Conclusions

This study externally validates our AMH-age based prediction of live birth for IVF.

The greatest utility of this external validation may be to confirm that AMH is an independent predictor of live birth. Moreover, as it was shown, AMH and age can be displayed as categories, rather continuous variables, with clear benefit for applying the model in a clinical environment.

However a couple’s predicted likelihood of live birth can range significantly, therefore it would require by the clinician interplay of both the interpretation of the nomogram results and individual patient evaluation.

## Abbreviations

AMH: Anti-Mullerian hormone; IVF: In vitro fertilization; ROC_AUC_: Area under the receiver operator curve; EV: External validation; BMI: Body mass index.

## Competing interests

Antonio La Marca, Scott Nelson and Richard Fleming have all received honorarium from Beckman Coulter and Roche Diagnostics to participate in Advisory Boards regarding AMH.

## Authors’ contributions

AK prepared the data and drafted the manuscript. SL and AM performed the statistical analysis and edited the manuscript. RF provided the clinical data and edited the manuscript. VL and AM developed the original nomogram and edited the manuscript. SN was responsive for the project, data integrity and final edit of the manuscript. All authors read and approved the final manuscript.

## Authors’ information

Antonio La Marca and Scott M Nelson joint senior authors.
